# Supplementary material for: Cell type-resolved connectomic reconstruction of the spinal dorsal horn reveals sensory neuron synaptic organization and modality-specific inhibition
Source: bioRxiv. 2026 Oct 1:2026.09.27.754691. Preprint. [Version 1] doi: 10.64898/2026.09.27.754691 (PMC13648474; doi:10.64898/2026.09.27.754691)
Supplement: Supplementary file 1 [file NIHPP2026.09.27.754691v1-supplement-1.pdf]

bioRxiv preprint doi: https://doi.org/10.64898/2026.09.27.754691; this version posted October 1, 2026. The copyright holder for this preprint (which was not certified by peer review) is the author/funder, who has granted bioRxiv a license to display the preprint in perpetuity. It is made available under a CC-BY 4.0 International license.

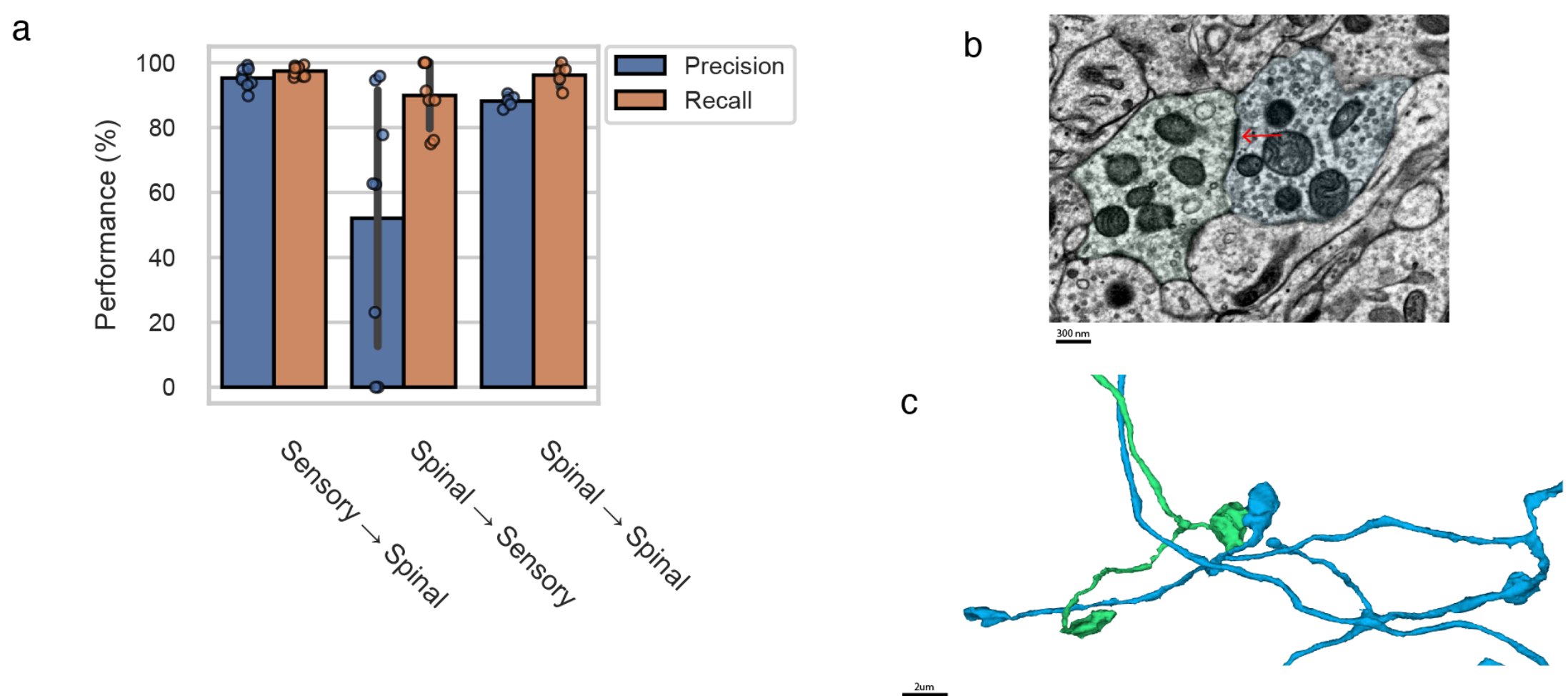

Figure S1 | Validation of synapse predictions

a, Precision and recall for different synapse-type predictions (**Methods**). The synapse prediction model has a low error rate when predicting axodendritic synapses formed by sensory neurons onto spinal cord neuron dendrites, as well as axodendritic synapses formed by spinal cord neurons onto other spinal cord neurons. However, it shows a higher false positive rate when predicting axoaxonic synapses made onto sensory neurons, especially for C-Cold neurons and peptidergic C-fiber neurons.

b, Example of a false positive axoaxonic synapse prediction on C-Cold neurons. Two adjacent C-Cold neurons form a symmetric membrane density that may confuse the synapse prediction model.

c, 3D view of the two adjacent C-Cold neurons.

bioRxiv preprint doi: https://doi.org/10.64898/2026.09.27.754691; this version posted October 1, 2026. The copyright holder for this preprint (which was not certified by peer review) is the author/funder, who has granted bioRxiv a license to display the preprint in perpetuity. It is made available under a CC-BY 4.0 International license.

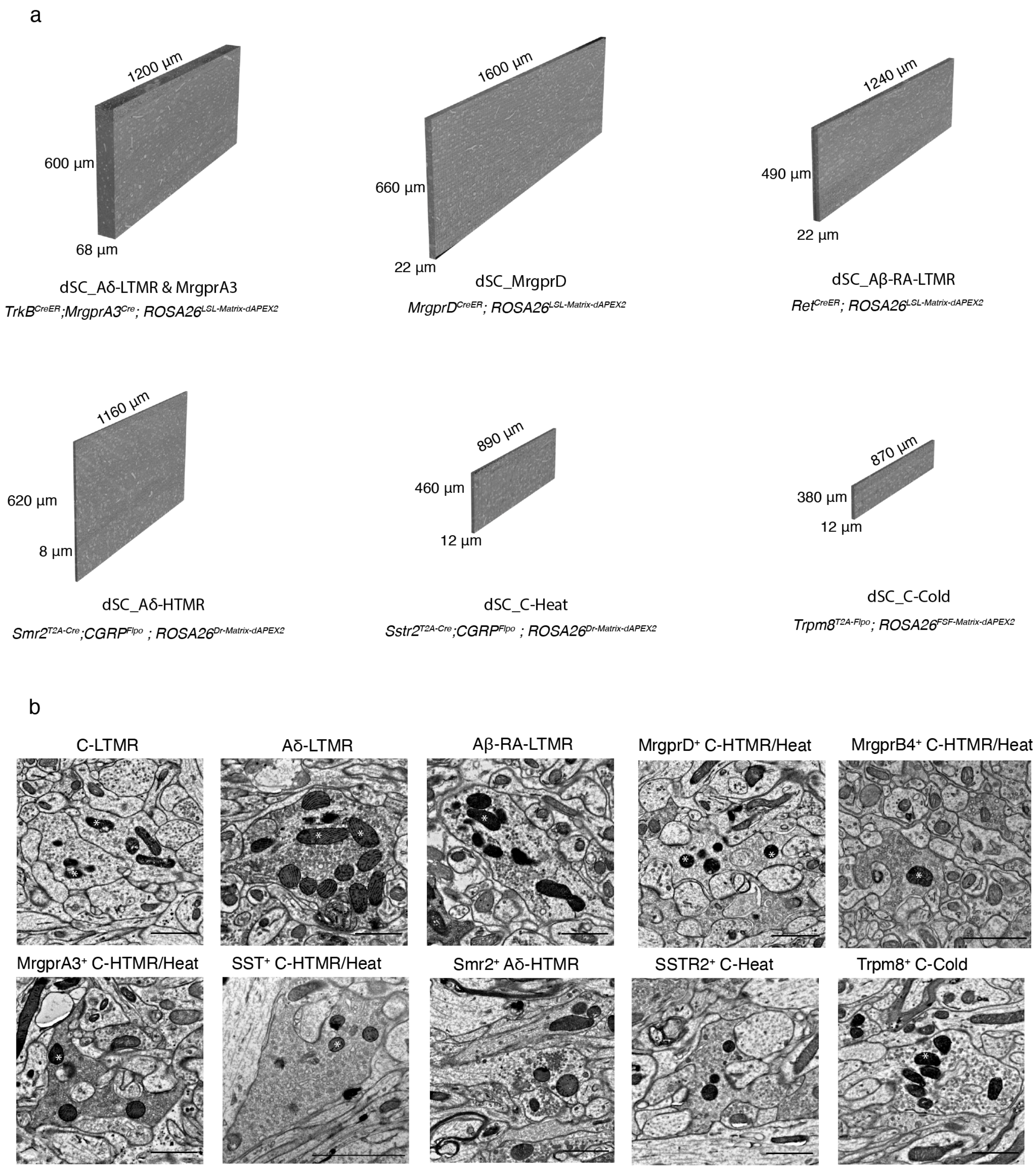

Figure S2 | dSC_APEX datasets with genetically labeled sensory neuron subtypes

a, Six EM volumes in which different sensory neuron subtypes are genetically labeled, illustrating the diversity of sensory inputs sampled in this study. C-LTMRs, Aβ-LTMRs, and MrgprD⁺ C-HTMR/Heat neurons were labeled by crossing a *ROSA26*$^{LSL\text{-}Matrix\text{-}dAPEX2}$ EM reporter line to *Th*$^{T2A\text{-}CreER}$, *Ret*^{CreER}, and *MrgprD*^{CreER} mice, respectively. Smr2⁺ Aδ-HTMRs (myelinated

bioRxiv preprint doi: https://doi.org/10.64898/2026.09.27.754691; this version posted October 1, 2026. The copyright holder for this preprint (which was not certified by peer review) is the author/funder, who has granted bioRxiv a license to display the preprint in perpetuity. It is made available under a CC-BY 4.0 International license.

mechano-nociceptors) and Sstr2^{+} C-Heat neurons (thermo-nociceptors) were labeled by crossing the $ROSA26^{DR\text{-}Matrix\text{-}dAPEX2}$ mouse line to $Smr2^{T2A\text{-}Cre}$*; CGRP*^{Flpo} and $Sstr2^{T2A\text{-}CreER}$*; CGRP*^{Flpo} mice, respectively. C-Cold neurons were labeled by crossing the $ROSA26^{FSF\text{-}Matrix\text{-}dAPEX2}$ reporter line to $Trpm8^{T2A\text{-}Flpo}$ mice. Note that Aδ-LTMRs and MrgprA3^{+} C-HTMR/Heat neurons were simultaneously labeled in $TrkB^{CreER}$*; MrgprA3*^{Cre}; $ROSA26^{LSL\text{-}Matrix\text{-}dAPEX2}$ mice. All dSC_APEX datasets were segmented except dSC_Aδ-HTMR, owing to imperfect image alignment caused by serial sectioning.

b, EM images of central terminals from genetically labeled sensory neurons in the lumbar spinal cord. MrgprB4^{+} and Sst^{+} C-HTMR/Heat neurons were labeled in single section TEM experiment using $MrgprB4^{Cre}$ and $Cysltr2^{Cre}$ animals harboring the $ROSA26^{DR\text{-}Matrix\text{-}dAPEX2}$ allele.

bioRxiv preprint doi: https://doi.org/10.64898/2026.09.27.754691; this version posted October 1, 2026. The copyright holder for this preprint (which was not certified by peer review) is the author/funder, who has granted bioRxiv a license to display the preprint in perpetuity. It is made available under a CC-BY 4.0 International license.

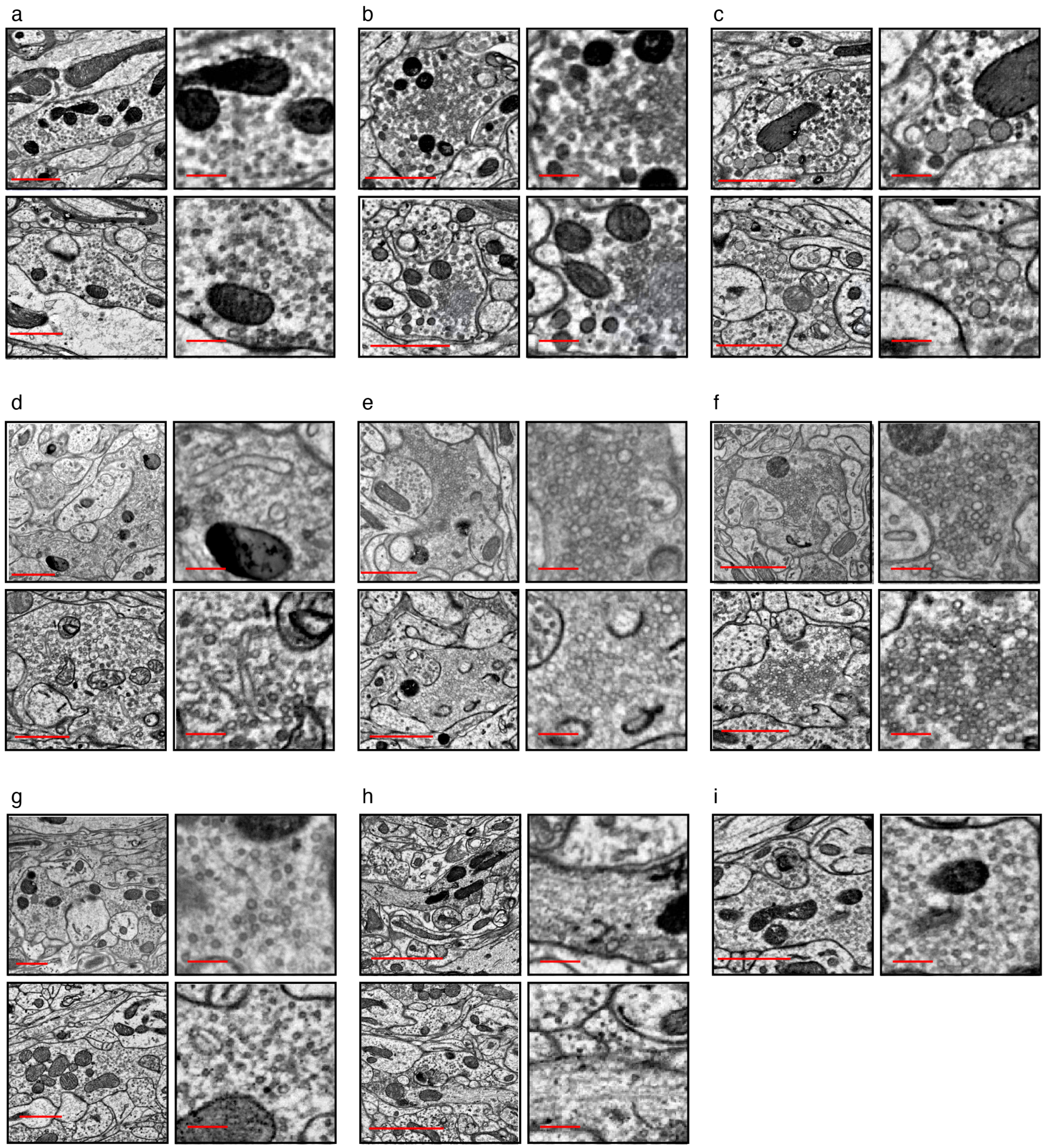

Figure S3 | Ultrastructural features of APEX-labeled sensory neurons and the ground truth for training SPINE

a, Trpm8⁺ C-Cold neuron morphologies. Top left, APEX labeled Trpm8⁺ C-Cold neuron in SC_C_Cold dataset. Top right, zoomed-in view of the same APEX labeled terminal shown in left. C-Cold terminals contain small, round, clear vesicles that are uniformly distributed within

bioRxiv preprint doi: https://doi.org/10.64898/2026.09.27.754691; this version posted October 1, 2026. The copyright holder for this preprint (which was not certified by peer review) is the author/funder, who has granted bioRxiv a license to display the preprint in perpetuity. It is made available under a CC-BY 4.0 International license.

the axonal bouton. Bottom left, C-Cold neuron identified in the dSC1 dataset that was used as ground truth for SPINE training. Bottom right, zoom in view of the same C-Cold neuron shown on the left.

b, Same as **a** but for peptidergic C-fiber neurons. Top, APEX labeled Sstr2$^+$ C-Nociceptor terminals contain dense-core vesicles as well as clusters of small clear vesicles. Bottom, a peptidergic C-fiber neuron identified in the dSC1 dataset similarly has dense core vesicles and is used as ground truth for SPINE training.

c, Same as **a** but for Aδ-HTMR. Top, Smr2$^+$ Aδ-HTMRs labeled by APEX contain large pale vesicles in terminals. Bottom, Aδ-HTMR ground truth annotated in the dSC1 dataset similarly have large pale vesicles.

d, Same as **a** but for Sst$^+$ C-HTMR/Heat neurons. Top, Sst$^+$ C-HTMR/Heat neurons labeled by APEX contain characteristic elongated vesicles. Bottom, Sst$^+$ C-HTMR/Heat ground truth annotated in the dSC1 dataset share similar ultrastructural features.

e, Top, MrgprA3$^+$ C-HTMR/Heat neurons labeled by APEX and zoomed-in view of the vesicles. Bottom, MrgprD$^+$ C-HTMR/Heat neurons labeled by APEX.

f, Top, MrgprB4$^+$ C-HTMR/Heat neurons labeled by APEX and zoomed-in view of the vesicles. Bottom, C-HTMR/Heat (MrgprD$^+$, MrgprA3$^+$, MrgprB4$^+$) ground truth annotated in the dSC1 dataset. C-HTMR/Heat neurons labeled by Mrgpr-family genes share similar ultrastructural features. They have fuzzy cytoplasm and clustered pale vesicles with variable sizes; we thus concatenated all three C-HTMR/Heat neurons as a single cluster.

g, Same as **a** but for Aδ-LTMR. All three LTMRs contain small round vesicles.

h, Same as **a** but for Aβ-LTMRs. Aβ-LTMR terminals contain small, round vesicles that are frequently clustered at presynaptic sites, and show abundant neurofilaments (right panels) in the axon terminals. Scale bar, left 2 µm; right, 250 nm.

i, C-LTMR ground truth examples used to train SPINE. C-LTMRs were APEX labeled in the dSC1 dataset. Scale bar for a-i left panels, 1 µm (except **h**); a-i, right panels, 250nm.

bioRxiv preprint doi: https://doi.org/10.64898/2026.09.27.754691; this version posted October 1, 2026. The copyright holder for this preprint (which was not certified by peer review) is the author/funder, who has granted bioRxiv a license to display the preprint in perpetuity. It is made available under a CC-BY 4.0 International license.

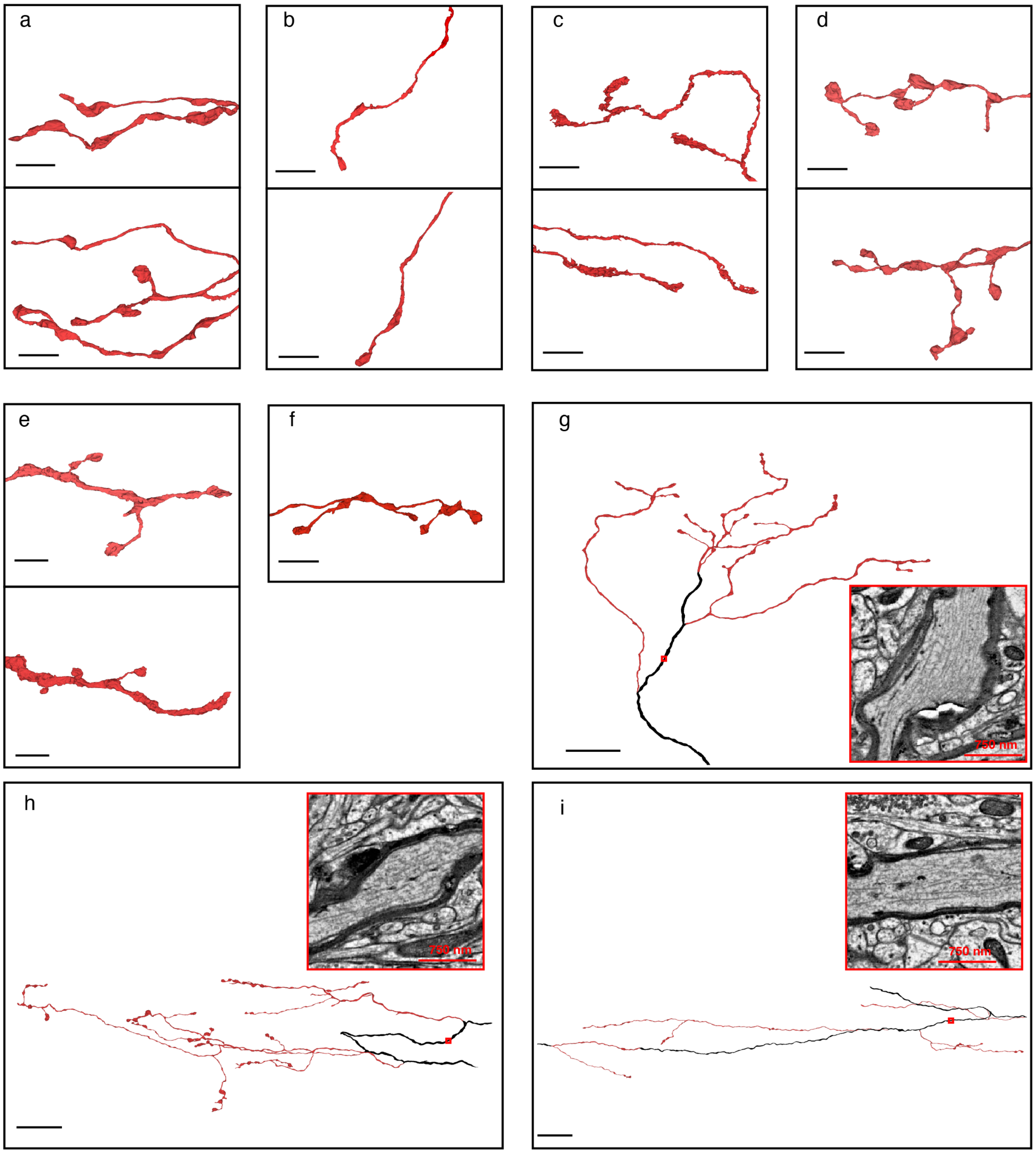

Figure S4 | Comparison of 3D morphologies of axonal boutons and cross-dataset validation

a, Top, 3D morphology of a Trpm8⁺ C-Cold neuron labeled by APEX. Bottom, C-Cold neuron axonal boutons identified in the dSC1 dataset that were used as ground truth for SPINE training. **b**, Same as **a** but for a peptidergic C-fiber neuron. Top, APEX labeled Sstr2⁺ C-Nociceptor terminals. Bottom, a peptidergic C-fiber neuron identified in the dSC1 dataset used as ground

bioRxiv preprint doi: https://doi.org/10.64898/2026.09.27.754691; this version posted October 1, 2026. The copyright holder for this preprint (which was not certified by peer review) is the author/funder, who has granted bioRxiv a license to display the preprint in perpetuity. It is made available under a CC-BY 4.0 International license.

truth for SPINE training.

c, Top, 3D morphology of a MrgprD^{+} C-HTMR/Heat neuron labeled by APEX. This neuron forms perforated axon terminals. Bottom, 3D morphology of a C-HTMR/Heat (MrgprD^{+}, MrgprA3^{+}, MrgprB4^{+}) ground truth neuron, this neuron similarly has an axon penetrated by postsynaptic components.

d, Same as **a** but for Aδ-LTMR. Aδ-LTMR axonal boutons are connected by thin neurites and form beads-on-a-string morphologies.

e, Same as **a** but for Aβ-LTMR.

f, 3D morphology of a C-LTMR ground truth neuron in the dSC1. Axonal boutons are connected by thin neurites and form beads-on-a-string morphologies.

g, Myelination pattern for Aβ-LTMRs in the dSC1 dataset. Aβ-LTMRs become unmyelinated (region outlined in red) to form synapses; the myelinated region is colored black. Zoomed-in view show myelin associated with this Aβ-LTMRs ground truth example and abundant neurofilament in the axons.

h, As in **g,** but for Aδ-LTMR ground truth in the dSC1 dataset. Aδ-LTMRs form beads-on-a-string bouton morphologies and contain fewer neurofilaments.

i, As in **g**, but for Aδ-HTMR ground truth in the dSC1 dataset. Aδ-HTMRs also contain neurofilaments in their axons. Scale bar **a-f**, 5 µm. **g-i**, 25µm.

bioRxiv preprint doi: https://doi.org/10.64898/2026.09.27.754691; this version posted October 1, 2026. The copyright holder for this preprint (which was not certified by peer review) is the author/funder, who has granted bioRxiv a license to display the preprint in perpetuity. It is made available under a CC-BY 4.0 International license.

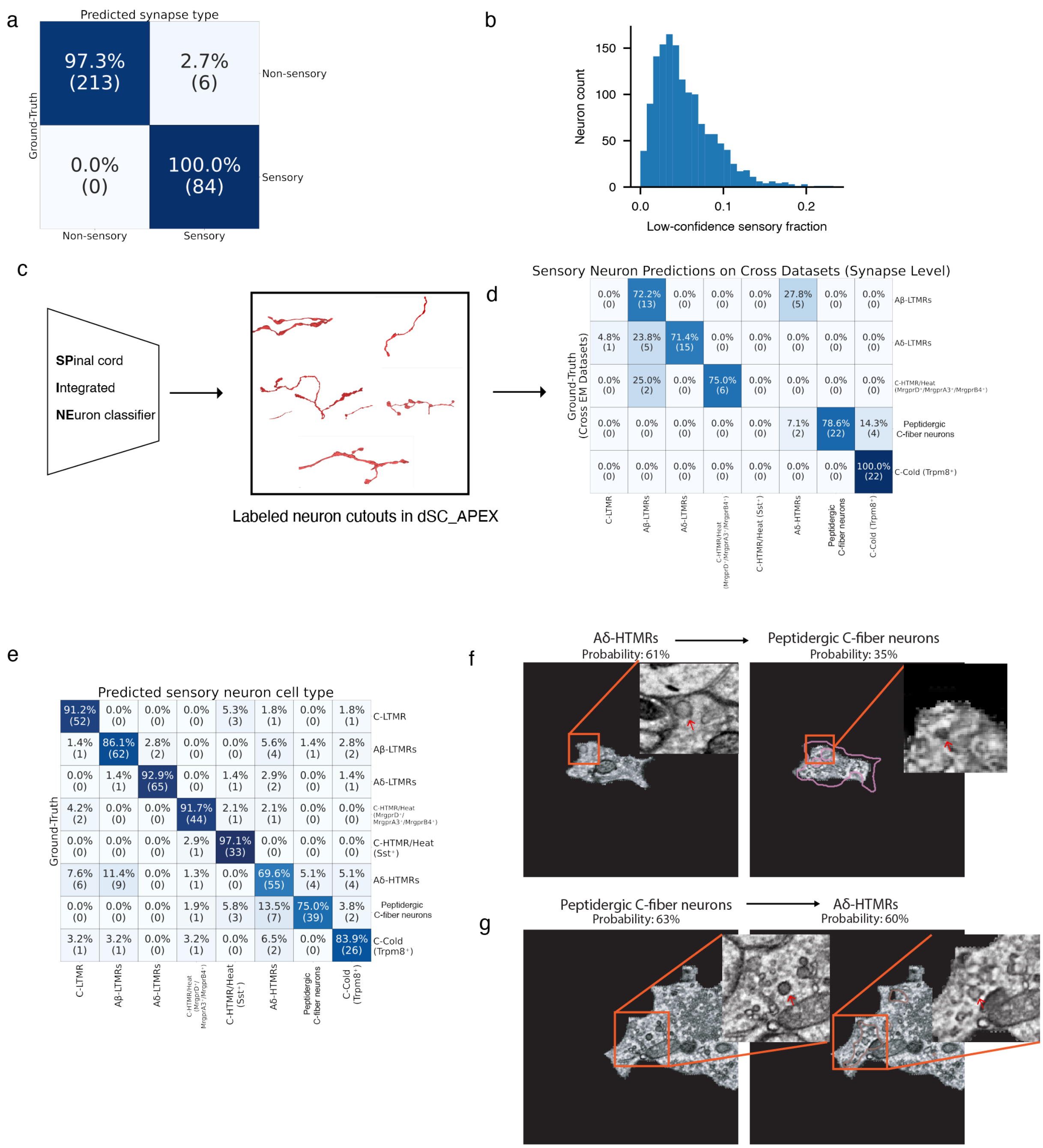

Figure S5 | SPINE validation and QuAC feature attributions

a, Confusion matrix showing SPINE classification accuracy in stage one: sensory versus non-sensory presynaptic site cutouts.

b, Distribution of low-confidence sensory predictions across 1,435 neurons classified by SPINE.

bioRxiv preprint doi: https://doi.org/10.64898/2026.09.27.754691; this version posted October 1, 2026. The copyright holder for this preprint (which was not certified by peer review) is the author/funder, who has granted bioRxiv a license to display the preprint in perpetuity. It is made available under a CC-BY 4.0 International license.

c, Schematic of cross-dataset validation. SPINE was trained on expert-annotated ground truth from the main dSC1 dataset and then evaluated on five additional APEX-labeled, segmented dSC_APEX EM volumes (**Figure S2a**) to assess cell-type generalization across datasets.

d, Confusion matrix showing SPINE classification accuracy across dSC_APEX datasets. SPINE was not applied to the dSC_Aδ-HTMR dataset because this dataset was not segmented.

e, Confusion matrix of a 2D classifier trained on dSC1 EM images of sensory neuron subtypes for QuAC.

f, Example of an Aδ-HTMR image (left) transformed by QuAC into a peptidergic C-fiber-like image (right). Zoomed-in views highlight altered pixels, including conversion of a large pale vesicle into a dark dense-core vesicle.

g, Reciprocal example showing a peptidergic C-fiber image (left) transformed into an Aδ-HTMR-like image (right), including conversion of a dark dense-core vesicle into a pale vesicle.

bioRxiv preprint doi: https://doi.org/10.64898/2026.09.27.754691; this version posted October 1, 2026. The copyright holder for this preprint (which was not certified by peer review) is the author/funder, who has granted bioRxiv a license to display the preprint in perpetuity. It is made available under a CC-BY 4.0 International license.

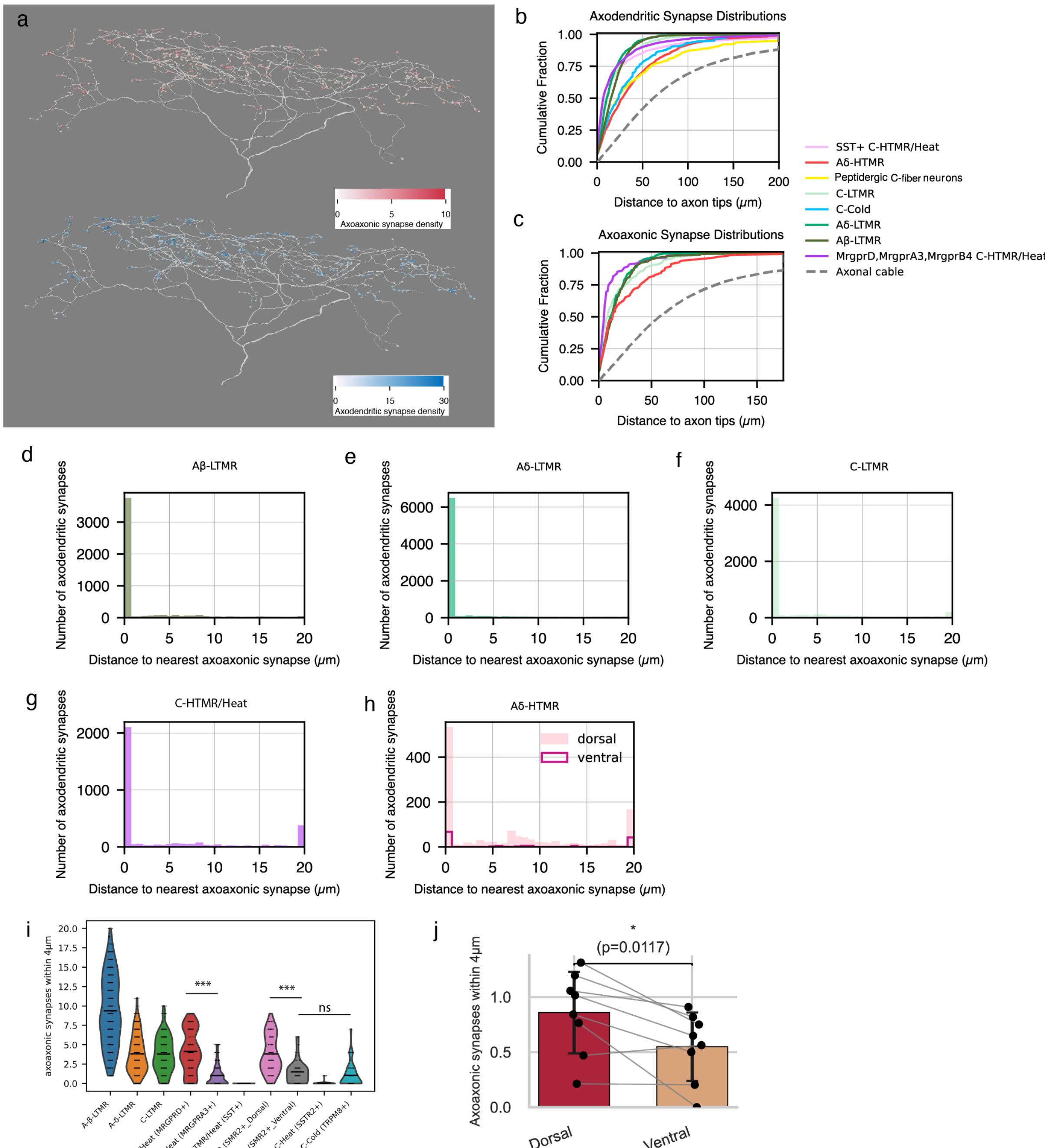

Figure S6| Distribution of axodendritic and axoaxonic synapses

a, Example Aδ-LTMR meshes color-coded by axoaxonic synapse density (top) or axodendritic synapse density (bottom).

b, Cumulative distributions of the cable-length distance from axodendritic synapses to the

bioRxiv preprint doi: https://doi.org/10.64898/2026.09.27.754691; this version posted October 1, 2026. The copyright holder for this preprint (which was not certified by peer review) is the author/funder, who has granted bioRxiv a license to display the preprint in perpetuity. It is made available under a CC-BY 4.0 International license.

nearest distal axon tip. Synapses are heavily distributed within the first 25μm from the axonal tips, gray curve shows the percentage of axons within certain distance from axonal tips. n = 10 neurons per subtype, except C-Cold (n = 9).

c, Cumulative distributions of the cable-length distance from validated axoaxonic synapses to the nearest distal axon tip. n = 2 axons per sensory subtype.

d–h, Histograms showing the cable-length distance from each axodendritic synapse to its nearest axoaxonic synapse. n = 2 axons per sensory subtype with manually verified axoaxonic synapses.

i, Number of axoaxonic synapses within 4 μm cable length of each axodendritic synapse in EM volumes in which sensory neurons were genetically labeled. Each dot represents one axodendritic synapse. At least five axons were sampled per genetically labeled sensory neuron subtype. $MrgprD^+$ C-HTMR/Heat versus $MrgprA3^+$ C-HTMR/Heat, $p = 6.38 \times 10^{-60}$; $Smr2^+$ Aδ-HTMR dorsal versus ventral terminals, $p = 5.41 \times 10^{-12}$; $Smr2^+$ Aδ-HTMR ventral terminals versus $Trpm8^+$ C-Cold, p = 1; Kruskal–Wallis test with Dunn's post hoc test. *n* = 42 Aβ-RA-LTMR, 102 Aδ-LTMR, 45 $MrgprD^+$ C-HTMR/Heat, 51 dorsal Aδ-HTMR, 79 C-LTMR, 45 ventral Aδ-HTMR, 42 C-Cold, 118 $MrgprA3^+$ C-HTMR/Heat, 34 $Sstr2^+$ C-Heat and 44 Sst^+ C-HTMR/Heat glomeruli.

j, Complete collateral arbors were reconstructed for 8 Aδ-HTMRs identified in the large EM volume, and the number of axoaxonic synapses within 4 μm cable length of each axodendritic synapse was quantified. Connected dots indicate dorsal and ventral branches of the same Aδ-HTMR. $p = 1.17 \times 10^{-2}$, Wilcoxon signed-rank test.

bioRxiv preprint doi: https://doi.org/10.64898/2026.09.27.754691; this version posted October 1, 2026. The copyright holder for this preprint (which was not certified by peer review) is the author/funder, who has granted bioRxiv a license to display the preprint in perpetuity. It is made available under a CC-BY 4.0 International license.

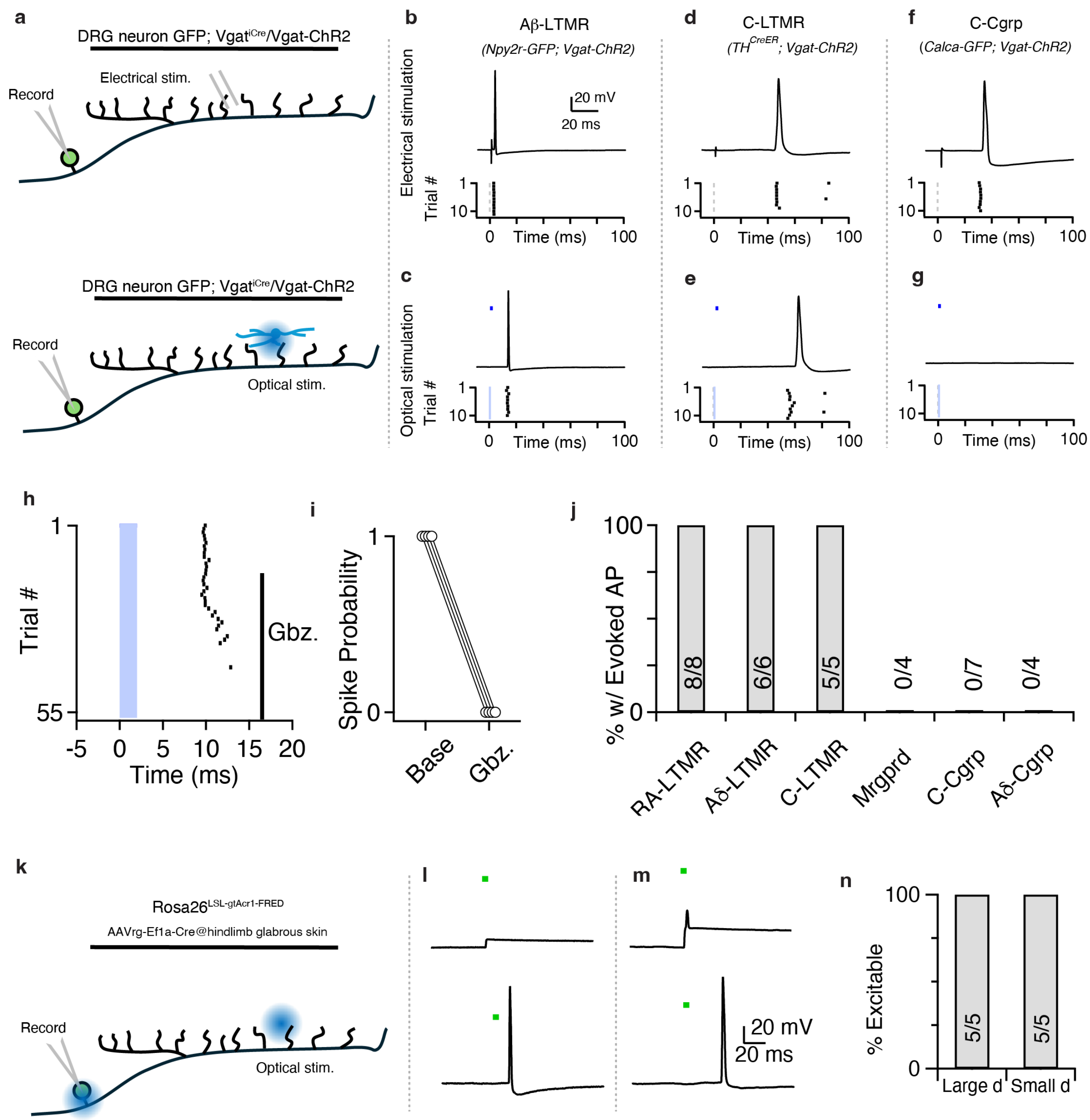

Figure S7| IhN activation generated back-propagated action potentials in LTMRs

a, Schematic of experimental setup. The spinal cord and attached DRGs were isolated ex vivo. Whole cell recordings were performed from DRG neuron subtypes identified with reporter mice

bioRxiv preprint doi: https://doi.org/10.64898/2026.09.27.754691; this version posted October 1, 2026. The copyright holder for this preprint (which was not certified by peer review) is the author/funder, who has granted bioRxiv a license to display the preprint in perpetuity. It is made available under a CC-BY 4.0 International license.

crossed with *Vgat-ChR2* or *Vgat-IRES-Cre; Rosa26LSL-ChR2* animals to express ChR2 in inhibitory interneurons. Electrical stimuli were delivered to the dorsal horn to confirm connectivity between DRG soma and central terminals, and to identify C, Aδ, and Aβ fibers (top). Optical stimuli were delivered to the dorsal horn through the microscope objective to excite inhibitory interneurons. Experiments were performed in the absence of fast excitatory transmission (AMPARs/NMDARs blocked) (bottom).

b, Example trace of an identified Aβ-LTMR with action potential back-propagated from the dorsal horn following electrical stimulation (top). Raster of 10 trials of electrical stimulation showing a consistency of the response and low jitter (bottom).

c, Same neuron as in **b**, but the action potential could be evoked by optical stimulation of dorsal horn inhibitory interneurons. Blue bar indicates light pulse timing.

d-e, Same as **b-c**, but for a C-LTMR.

f-g, Same as **b-c**, but for a CGRP-positive C-fiber. Optical activation anywhere in the spinal cord failed to evoke back-propagating action potentials.

h, Example Aβ-LTMR with action potentials evoked by activation of dorsal horn inhibitory interneurons. Addition of SR95531 (gabazine, Gbz.) to the bath to inhibit GABAARs eliminated evoked action potentials.

i, Probability of evoking action potentials from optical stimulation of the spinal cord at baseline and after the addition of SR95531 to block GABAARs in four Aβ-LTMRs.

j, Fraction of neurons in which optical activation of dorsal horn inhibitory interneurons could evoke an action potential for identified subtypes of DRG neurons. Numbers indicate number of neurons.

k, Schematic of Acr1 excitation in DRG neurons. Same preparation as shown in a, but for animals expressing the light-activated chloride channel Acr1. Hindlimb glabrous skin of Rosa26-LSL-gtAcr1-FRED animals was injected with AAVrg-Cre. Optical stimuli were delivered to the dorsal horn or cell body of randomly recorded DRG neurons.

l, Putative Aβ-LTMR (identified by A-fiber conduction velocity) with optical activation of the cell body (top) and optical activation of dorsal horn terminals (bottom). Green bar indicates light pulse timing.

m, Same as in l, but for a C-HTMR (identified by C-fiber conduction velocity and C-HTMR spiking pattern).

bioRxiv preprint doi: https://doi.org/10.64898/2026.09.27.754691; this version posted October 1, 2026. The copyright holder for this preprint (which was not certified by peer review) is the author/funder, who has granted bioRxiv a license to display the preprint in perpetuity. It is made available under a CC-BY 4.0 International license.

n, Fraction of large (A-fiber) and small (C-fiber) neurons that could be excited by optical activation of dorsal horn terminals. Numbers indicate number of neurons.

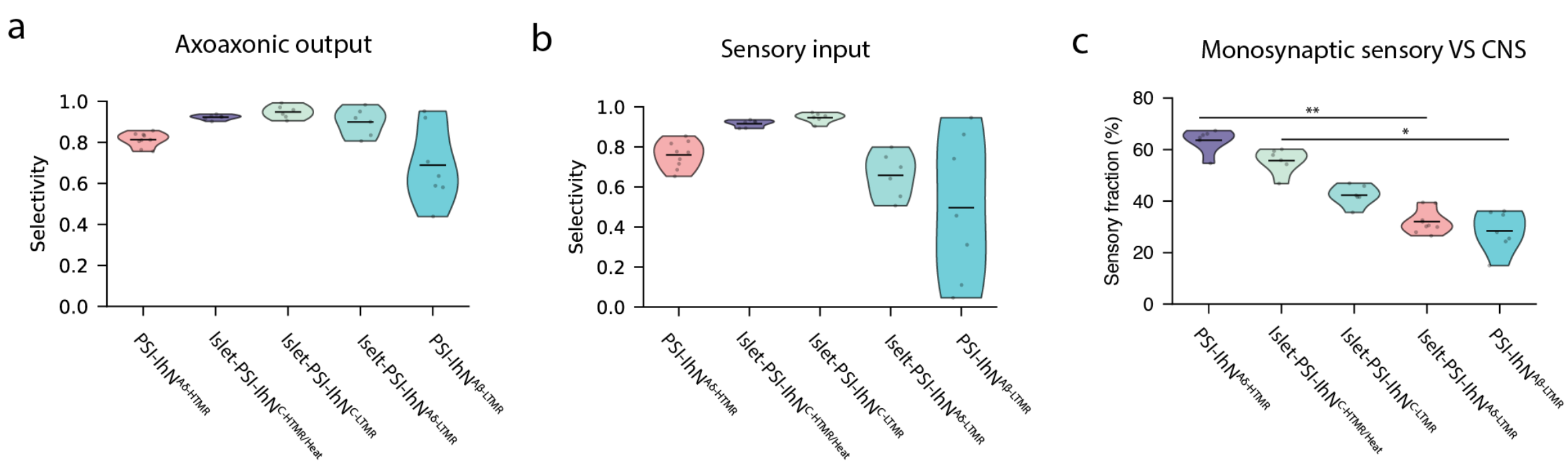

Figure S8| Homotypic feedback inhibition (PSI) mediated by PSI-IhNs

a, Fraction of sensory input from the corresponding sensory subtype received by each PSI-IhN population. For example, for Islet-PSI-IhN$^{C\text{-}LTMR}$ neurons, selectivity is defined as the fraction of C-LTMR input among all sensory inputs to the neuron.

b, Fraction of axoaxonic outputs from each PSI-IhN population directed onto the corresponding sensory subtype.

c, Fraction of direct sensory versus non-sensory (CNS) synaptic inputs onto each PSI-IhN population. (Islet-PSI-IhN$^{C\text{-}HTMR/Heat}$ versus PSI-IhN$^{A\delta\text{-}LTMR}$, $p=2.68\times10^{-4}$, Islet-PSI-IhN$^{C\text{-}LTMR}$ versus PSI-IhN$^{A\beta\text{-}LTMR}$, $p=7.04\times10^{-3}$, Kruskal-Wallis test, Dunn post hoc test).

bioRxiv preprint doi: https://doi.org/10.64898/2026.09.27.754691; this version posted October 1, 2026. The copyright holder for this preprint (which was not certified by peer review) is the author/funder, who has granted bioRxiv a license to display the preprint in perpetuity. It is made available under a CC-BY 4.0 International license.

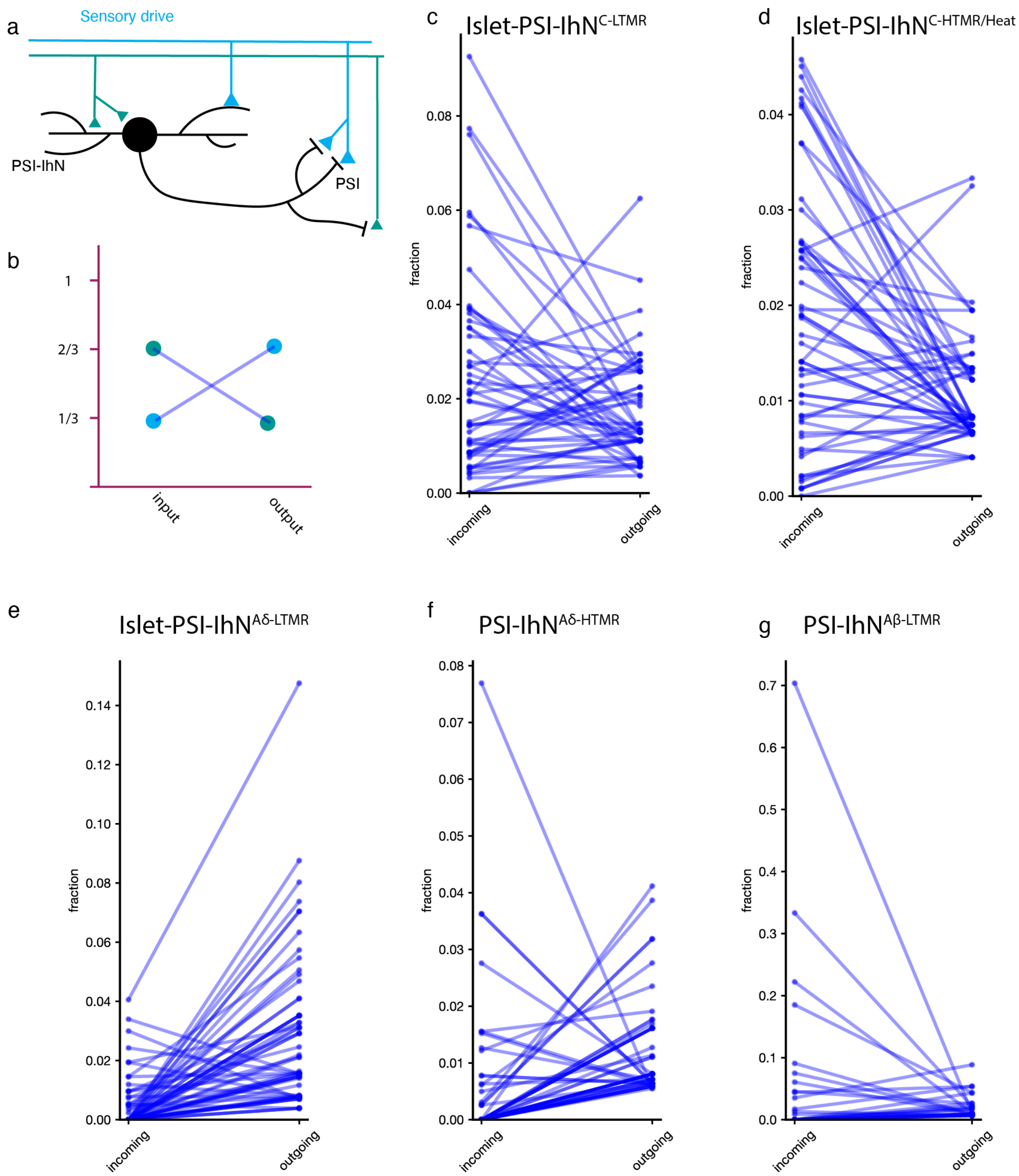

Figure S9 | Lateral inhibition mediated by PSI-IhNs

a, Schematic of sensory neurons that both drive and receive PSI. A sensory neuron can excite a GABAergic spinal cord neuron that in turn forms axoaxonic synapses onto the same sensory

bioRxiv preprint doi: https://doi.org/10.64898/2026.09.27.754691; this version posted October 1, 2026. The copyright holder for this preprint (which was not certified by peer review) is the author/funder, who has granted bioRxiv a license to display the preprint in perpetuity. It is made available under a CC-BY 4.0 International license.

neuron (self-inhibition) or onto other sensory neurons (lateral inhibition).

b, Quantification of incoming and outgoing fractions for the two example sensory neurons in **a**. The green sensory neuron contributes 2/3 of the sensory input onto the GABAergic neuron and receives 1/3 of its outgoing axoaxonic synapses. The light blue sensory neuron contributes 1/3 of the sensory input and receives 2/3 of the axoaxonic synapses.

c, Incoming and outgoing fractions for C-LTMR and islet-PSI-IhN$^{\text{C-LTMR}}$ pairs. 52 C-LTMR–islet-PSI-IhN$^{\text{C-LTMR}}$ pairs were analyzed.

d, Incoming and outgoing fractions for C-HTMR/Heat and islet-PSI-IhN$^{\text{C-HTMR/Heat}}$ pairs. 55 pairs were analyzed.

e, Incoming and outgoing fractions for Aδ-LTMR and Islet-PSI-IhN$^{\text{A}\delta\text{-LTMR}}$ pairs. 60 pairs were analyzed.

f, Incoming and outgoing fractions for Aδ-HTMR and PSI-IhN$^{\text{A}\delta\text{-HTMR}}$ pairs. 49 pairs were analyzed.

g, Incoming and outgoing fractions for Aβ-LTMR and PSI-IhN$^{\text{A}\beta\text{-LTMR}}$ pairs. 31 pairs were analyzed.

bioRxiv preprint doi: https://doi.org/10.64898/2026.09.27.754691; this version posted October 1, 2026. The copyright holder for this preprint (which was not certified by peer review) is the author/funder, who has granted bioRxiv a license to display the preprint in perpetuity. It is made available under a CC-BY 4.0 International license.

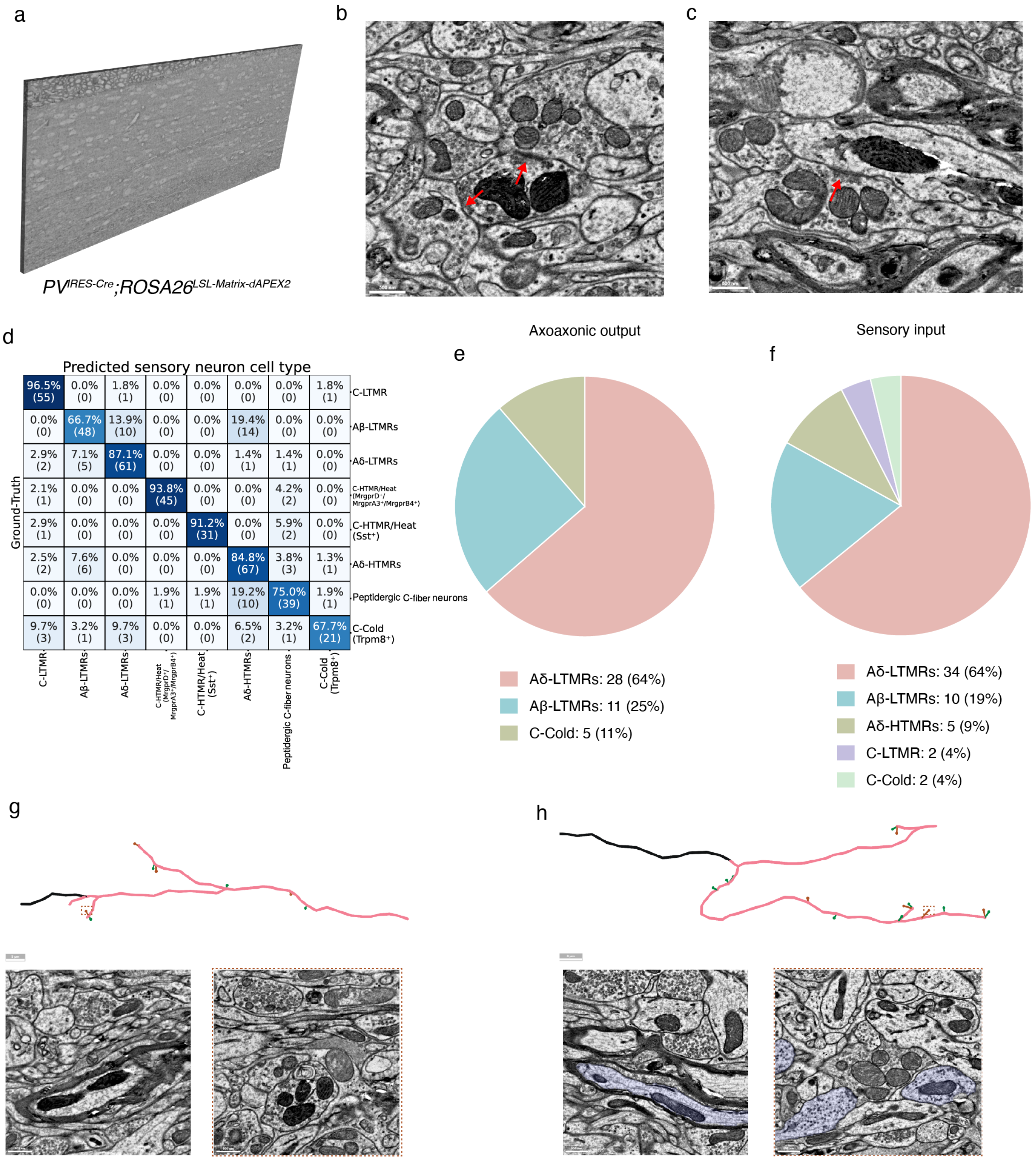

Figure S10 | PV^{+} PSI-IhNs form axoaxonic synapses onto A-fiber LTMRs

a, An EM volume in which PV^{+} neurons were labeled. The dataset consists of 210 serial-section EM images from a *PV$^{IRES\text{-}Cre}$; ROSA26$^{LSL\text{-}Matrix\text{-}dAPEX2}$* mouse.

bioRxiv preprint doi: https://doi.org/10.64898/2026.09.27.754691; this version posted October 1, 2026. The copyright holder for this preprint (which was not certified by peer review) is the author/funder, who has granted bioRxiv a license to display the preprint in perpetuity. It is made available under a CC-BY 4.0 International license.

b, APEX-labeled PV⁺ axon forming two axoaxonic synapses onto sensory neurons (red arrows).

c, Sensory neuron forming axodendritic synapses onto an APEX-labeled PV⁺ dendrite (red arrow).

d, Confusion matrix of the classifier trained on 4 nm resolution 2D EM images for sensory neuron subtype prediction.

e, Predicted subtypes of 44 randomly sampled sensory neurons postsynaptic to PV⁺ axons. Most axoaxonic outputs are classified as A-LTMRs, with the majority identified as Aδ-LTMRs.

f, Same as **e**, but for 53 randomly sampled sensory neurons presynaptic to PV⁺ dendrites. Most inputs are predicted to arise from A-LTMRs, with Aδ-LTMRs comprising the largest fraction.

g, PV⁺ PSI-IhNs have myelinated axons. Top, skeleton trace of a PV⁺ PSI-IhN axon fragment from the $PV^{IRES\text{-}Cre}$; $ROSA26^{LSL\text{-}Matrix\text{-}dAPEX2}$ dataset. Black segments indicate myelinated portions of the axon, and pink segments indicate unmyelinated regions. Brown dots mark axoaxonic synapses, and green dots mark axodendritic synapses. Bottom left, EM image showing myelin wrapping the axon. Bottom right, EM image showing an axoaxonic synapse formed by this neuron.

h, Same as **g**, but from the C-LTMR-labeled dSC1 dataset, showing a myelinated Islet-PSI-IhN$^{A\delta\text{-}LTMR}$ neuron.

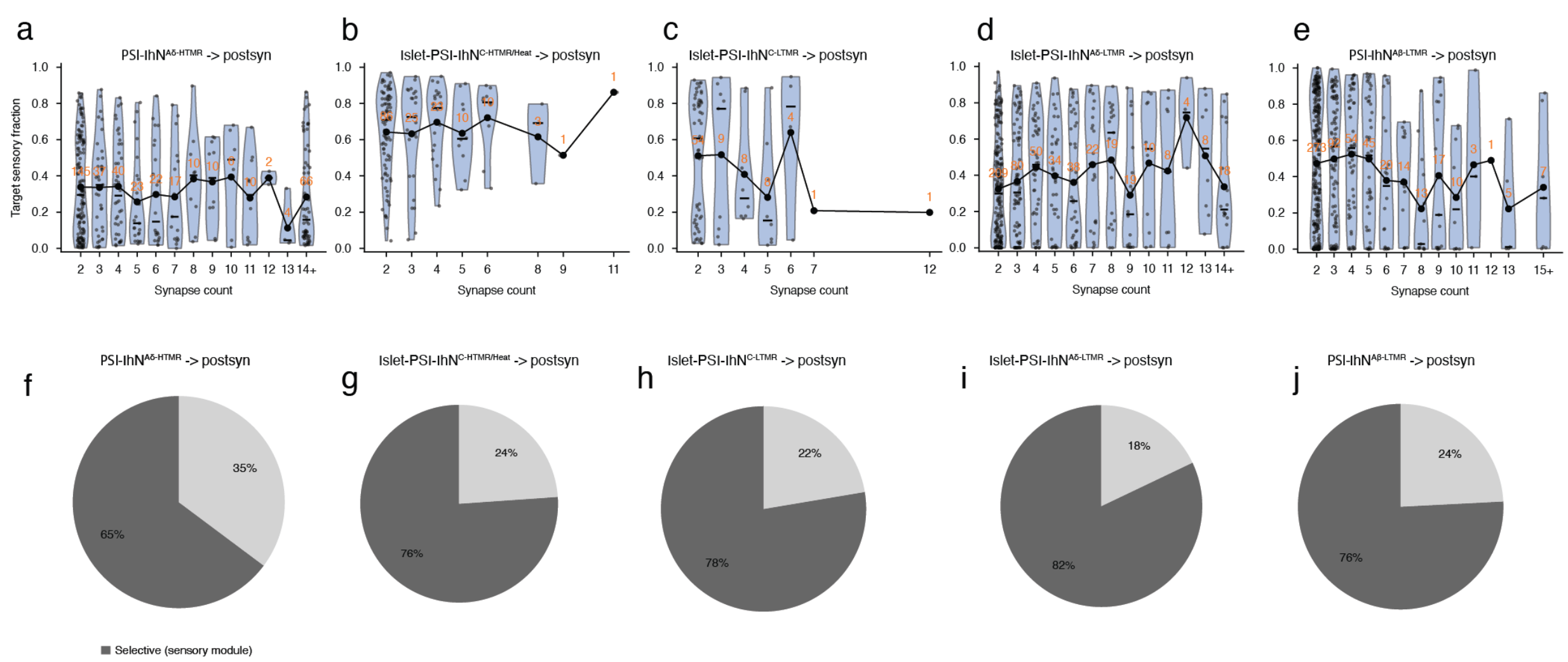

bioRxiv preprint doi: https://doi.org/10.64898/2026.09.27.754691; this version posted October 1, 2026. The copyright holder for this preprint (which was not certified by peer review) is the author/funder, who has granted bioRxiv a license to display the preprint in perpetuity. It is made available under a CC-BY 4.0 International license.

Figure S11| Heterotypic postsynaptic inhibition mediated by PSI-IhNs

a–e, Fraction of sensory input from the corresponding sensory subtype onto postsynaptic neurons of each PSI-IhN population targeted by axodendritic synapses. For example, for postsynaptic targets of Islet-PSI-IhN$^{\text{C-LTMR}}$ neurons, this value is the fraction of C-LTMR input among all sensory inputs to the postsynaptic neuron. Data are plotted as a function of the number of synapses between the PSI-IhN neuron and the postsynaptic target. Yellow numbers indicate neuron counts. (n=392 for PSI-IhN$^{\text{A}\delta\text{-HTMR}}$ postsynaptic neurons, n=159 for Islet-PSI-IhN$^{\text{C-HTMR/Heat}}$ postsynaptic neurons, n=85 for Islet-PSI-IhN$^{\text{C-LTMR}}$ postsynaptic neurons, n=519 for Islet-PSI-IhN$^{\text{A}\delta\text{-LTMR}}$ postsynaptic neurons, n=554 for PSI-IhN$^{\text{A}\beta\text{-LTMR}}$ postsynaptic neurons.)
f–j, Fraction of postsynaptic neurons targeted by axodendritic synapses assigned to specific sensory modules, defined as neurons receiving more than 50% of their sensory input from a single sensory subtype.

bioRxiv preprint doi: https://doi.org/10.64898/2026.09.27.754691; this version posted October 1, 2026. The copyright holder for this preprint (which was not certified by peer review) is the author/funder, who has granted bioRxiv a license to display the preprint in perpetuity. It is made available under a CC-BY 4.0 International license.

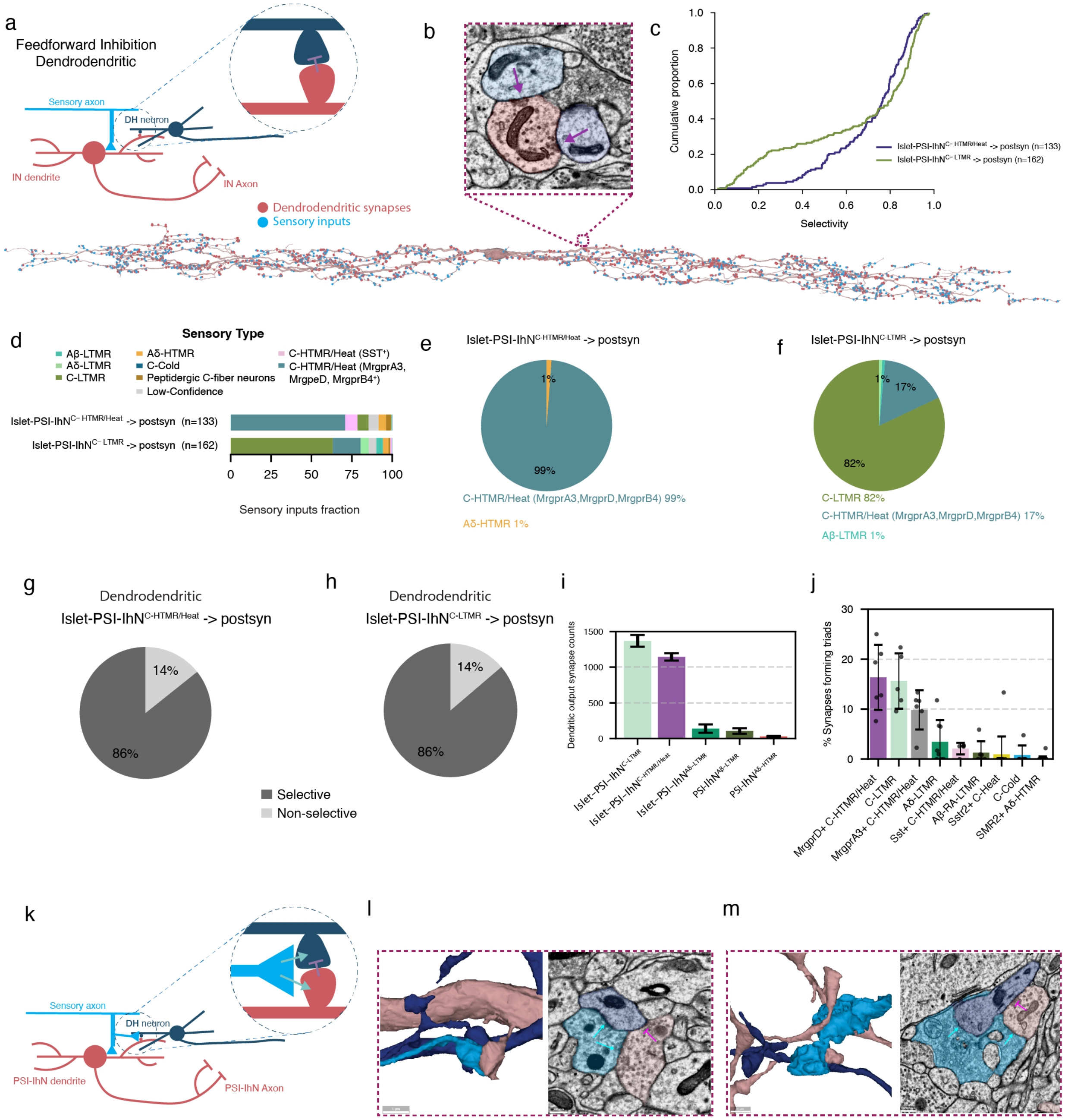

Figure S12| Dendrodendritic synapses mediated by PSI-IhNs

a, Schematic of sensory-evoked feedforward inhibition mediated by dendrodendritic synapses.

bioRxiv preprint doi: https://doi.org/10.64898/2026.09.27.754691; this version posted October 1, 2026. The copyright holder for this preprint (which was not certified by peer review) is the author/funder, who has granted bioRxiv a license to display the preprint in perpetuity. It is made available under a CC-BY 4.0 International license.

b, Example Islet-PSI-IhN$^{\text{C-HTMR/Heat}}$ neuron showing sensory input sites and dendrodendritic output synapses. Insets show a dendritic spine forming two dendrodendritic synapses.

c, Cumulative distributions of the homotypic selectivity index for all postsynaptic neurons targeted by dendrodendritic synapses. Numbers of PSI-IhN–postsynaptic neuron connections are indicated.

d, Sensory input fractions of postsynaptic neuron populations targeted by dendrodendritic synapses from Islet-PSI-IhN$^{\text{C-LTMR}}$ and Islet-PSI-IhN$^{\text{C-HTMR/Heat}}$ neurons.

e,f, Fractions of sensory modules represented by postsynaptic neurons of each PSI-IhN population targeted by dendrodendritic synapses.

g,h, Fraction of postsynaptic neurons targeted by dendrodendritic synapses assigned to specific sensory modules.

i, Number of dendritic output synapses formed by PSI-IhN neurons.

j, Fraction of axodendritic synapses within each glomerulus that are part of triadic synaptic arrangements.

k, Schematic of a triadic synapse, in which a sensory bouton contacts the dendrites of two spinal neurons, one of which then forms a dendrodendritic synapse onto the other.

l, Example triad formed between a C-LTMR bouton and an Islet-PSI-IhN$^{\text{C-LTMR}}$ neuron. Axodendritic synapses from sensory neurons are annotated as cyan arrows, dendrodendritic synapses from Islet neurons are annotated as purple line.

m, same as **i**, example triad formed between a C-HTMR/Heat bouton and an Islet-PSI-IhN$^{\text{C-HTMR/Heat}}$ neuron.

bioRxiv preprint doi: https://doi.org/10.64898/2026.09.27.754691; this version posted October 1, 2026. The copyright holder for this preprint (which was not certified by peer review) is the author/funder, who has granted bioRxiv a license to display the preprint in perpetuity. It is made available under a CC-BY 4.0 International license.

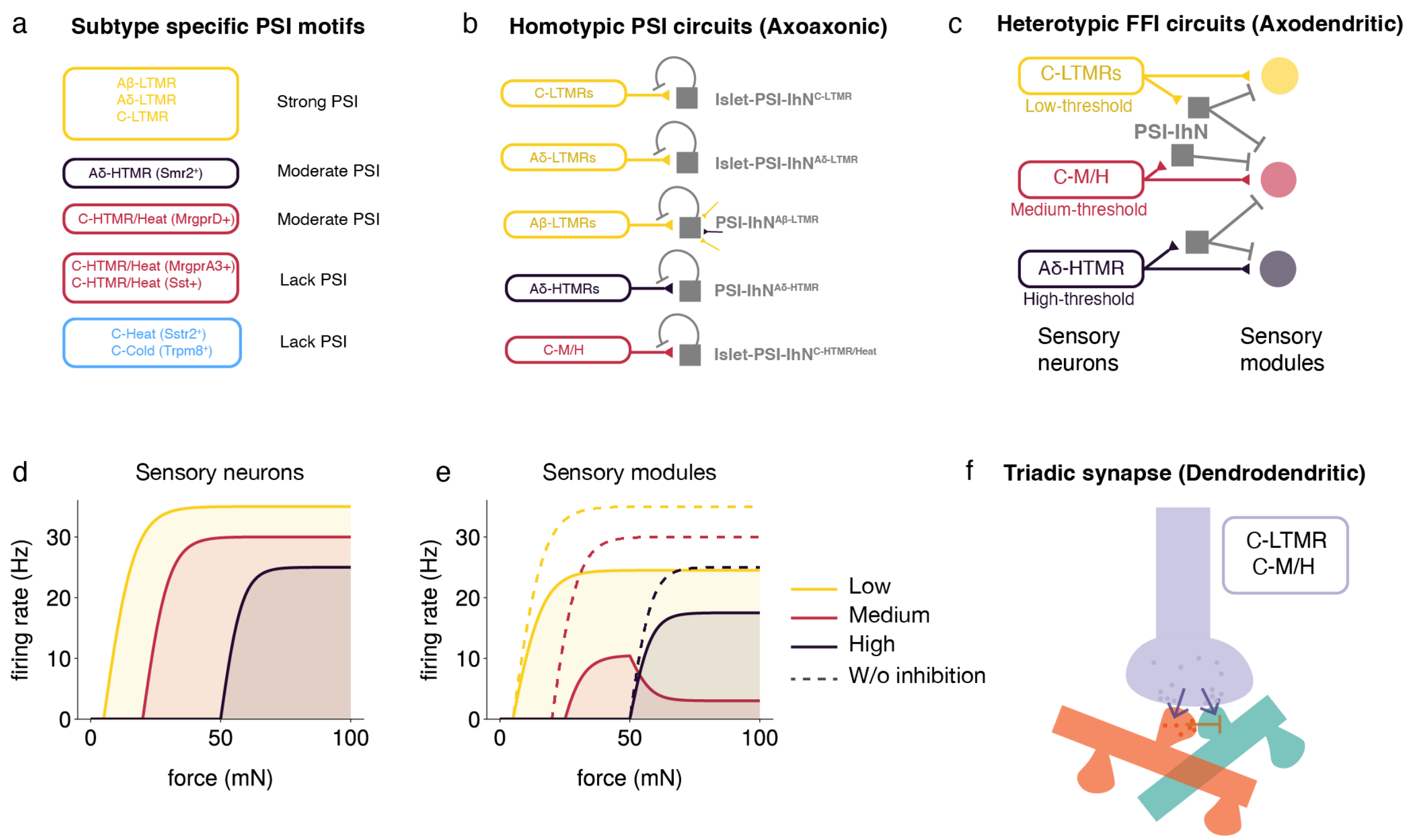

Figure S13 | Summary of sensory neuron subtype-evoked feedback and feedforward inhibition motifs of the superficial dorsal horn

a, b, Schematic of homotypic feedback inhibition across sensory neuron subtypes. Aβ-LTMRs, Aδ-LTMRs, and C-LTMRs strongly recruit their corresponding PSI-IhN populations and, in turn, receive abundant axoaxonic input from these GABAergic neurons. Aβ-LTMRs also receive a smaller degree of heterotypic PSI recruited by other LTMR subtypes and Aδ-HTMRs. Aδ-HTMR nociceptors and MrgprD^{+} C-HTMR/Heat neurons receive intermediate levels of homotypic feedback inhibition, whereas C-Cold and C-Heat thermoreceptors and Sst^{+} and MrgprA3^{+} C-HTMR/Heat neurons receive relatively sparse axoaxonic input.

c, Schematic of the heterotypic FFI circuit. C-LTMRs and Aδ-HTMR both inhibit C-HTMR/Heat module through corresponding PSI-IhN populations. This heterotypic inhibition can potentially sharpen modality tuning, as simulated in **d,e**.

bioRxiv preprint doi: https://doi.org/10.64898/2026.09.27.754691; this version posted October 1, 2026. The copyright holder for this preprint (which was not certified by peer review) is the author/funder, who has granted bioRxiv a license to display the preprint in perpetuity. It is made available under a CC-BY 4.0 International license.

d, Simulated force-tuning curves of C-LTMRs, C-HTMR/Heat neurons, and Aδ-HTMRs , based on data from (Qi et al., 2024); (Lezgiyeva et al., 2026); (Ma et al., 2012); and (Pitcher et al., 2016).

e, Simulated force-tuning curves of postsynaptic neurons in each sensory module, with and without feedforward inhibition. Without inhibition (dashed curves), postsynaptic tuning largely mirrors presynaptic sensory tuning. With feedforward inhibition (solid curves), the C-HTMR/Heat module shifts to higher response thresholds, loses responses in the LTMR range, and exhibits reduced firing in the nociceptor range.

f, Schematic of triadic synapses uniquely formed between C-LTMRs or C-HTMR/Heat neurons and their corresponding Islet-PSI-IhN populations.
